# Utilizing insights of DNA repair machinery to discover MMEJ deletions and novel mechanisms

**DOI:** 10.1093/nar/gkae1132

**Published:** 2024-11-28

**Authors:** Aditee Kadam, Shay Shilo, Hadas Naor, Alexander Wainstein, Yardena Brilon, Tzah Feldman, Mark Minden, Nathali Kaushansky, Noa Chapal-Ilani, Liran Shlush

**Affiliations:** Department of Molecular Cell Biology, Weizmann Institute of Science, Rehovot 761001, Israel; Department of Molecular Cell Biology, Weizmann Institute of Science, Rehovot 761001, Israel; Department of Molecular Cell Biology, Weizmann Institute of Science, Rehovot 761001, Israel; Department of Molecular Cell Biology, Weizmann Institute of Science, Rehovot 761001, Israel; Sequentify Ltd., 10 Moti Kind St., 5th Floor, Rehovot 7638519, Israel; Department of Molecular Cell Biology, Weizmann Institute of Science, Rehovot 761001, Israel; Princess Margaret Cancer Centre, University Health Network (UHN), Department of Medical Oncology & Hematology, Toronto, ON M5G 2C4, Canada; Department of Molecular Cell Biology, Weizmann Institute of Science, Rehovot 761001, Israel; Department of Molecular Cell Biology, Weizmann Institute of Science, Rehovot 761001, Israel; Department of Molecular Cell Biology, Weizmann Institute of Science, Rehovot 761001, Israel; Molecular Hematology Clinic Maccabi Healthcare Services, Tel Aviv 6812509, Israel

## Abstract

We developed Del-read, an algorithm targeting medium-sized deletions (6–100 bp) in short-reads, which are challenging for current variant callers relying on alignment. Our focus was on Micro-Homolog mediated End Joining deletions (MMEJ-dels), prevalent in myeloid malignancies. MMEJ-dels follow a distinct pattern, occurring between two homologies, allowing us to generate a comprehensive list of MMEJ-dels in the exome. Using Del-read, we identified numerous novel germline and somatic MMEJ-dels in BEAT-AML and TCGA-breast datasets. Validation in 672 healthy individuals confirmed their presence. These novel MMEJ-dels were linked to genomic features associated with replication stress, like G-quadruplexes and minisatellite. Additionally, we observed a new category of MMEJ-dels with an imperfect-match at the flanking sequences of the homologies, suggesting a mechanism involving mispairing in homology alignment. We demonstrated robustness of the repair system despite CRISPR/Cas9-induced mismatches in the homologies. Further analysis of the canonical *ASXL1* deletion revealed a diverse array of these imperfect-matches. This suggests a potentially more flexible and error-prone MMEJ repair system than previously understood. Our findings highlight Del-read's potential in uncovering previously undetected deletions and deepen our understanding of repair mechanisms.

## Graphical Abstract

**Graphical Abstract:**
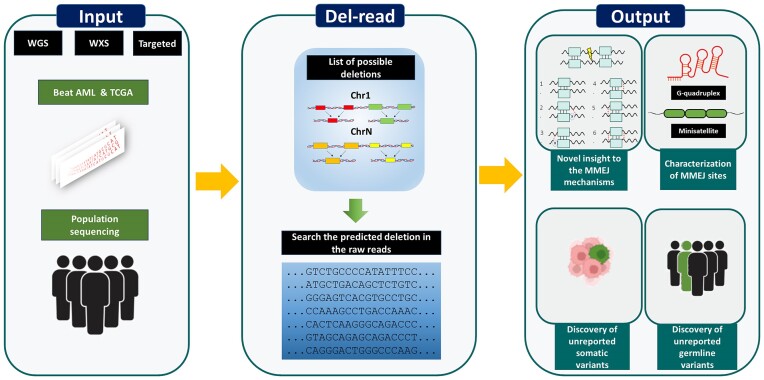

## Introduction

Deletions are a fundamental genetic feature that is associated with numerous phenotypes. Although deletions have been explored since the early days of modern genetics, recent research and advances, especially in long-read sequencing technologies, demonstrated that their detection is not optimal (1–5). Current deletion detection methods are primarily based on aligning short reads to a reference genome, and due to mapping misinterpretation, they can fail to identify deletions (6). Consequently, predictions from different deletion callers are often inconsistent, and this inconsistency is most significant for the medium-sized indels (50–100 bp) (3). Usually, deletions are formed as a result of DNA repair after double-strand breaks (DSBs) (7–11). Different repair mechanisms generate a unique molecular signature around the breakpoints of the deletion (12,13). MMEJ deletions (MMEJ-dels), the most common deletions in myeloid malignancies (14), have a clear, defined and predictable signature called ‘homologies’ owing to a deterministic repair mechanism. Homologies are short, identical sequences in close proximity. MMEJ repair utilizes homologies at the flanking sequences of DSBs, leaving behind just one homolog sequence after the deletion (15).

We designed a novel algorithm, Del-read, that utilizes the predictability of the deterministic outcome of MMEJ-dels. The algorithm is built on a precompiled dataset of ‘predicted deletions’. When reads from whole genome or exome sequencing are fed to the algorithm, the dataset of predicted deletions is compared directly with the reads. Such a concept can be extended to look for any possible indels in a specific region or in the entire genome if computational resources are not limited. Accordingly, we applied the predictions to two states: (i) all the possible deletions in the DSB hotspot region of *ASXL1*/*SRSF2* genes and (ii) all the possible MMEJ-dels based on the identification of all homologies (in a window of 100 bp) in the exome. As proof of principle, we applied our pipeline to the paired tumor and normal control samples of BEAT-AML (16) and TCGA-BRCA (17) datasets and found 425 novel germline and 20 novel somatic MMEJ-dels. Our analysis revealed that MMEJ-dels were enriched in minisatellite and G-quadruplexes, suggesting a potential DNA secondary structure-dependent deletion formation. Finally, we demonstrate that MMEJ repair is robust to mismatches, and solidify imperfect-match deletion mechanism through greedy pairing of the homologs.

## Materials and methods

### Samples

Sequence data of 359 paired tumor and control samples aligned to the reference hg19 were obtained from BEAT-AML. Additionally, 200 paired and 225 unpaired WES breast cancer samples from the TCGA aligned to GRCh38.d1.vd1.fa were obtained.

### Variant calling pipeline

Deletions were called using three variant callers - Varscan2 (2.3.9), Platypus (0.8.1) and Delly (0.7.6) with default parameters. Varscan uses mapped reads and then calls indels using the aligned data and some filtering steps. Platypus uses read alignments, local assembly of small regions to identify variants. Delly uses a combination of strategies: paired-ends consensus and split-read where the unaligned reads are split and attempted to align it in two portions.

To specifically identify MMEJ-dels from the total deletions, homologies were probed around the deletions. The minimum size of homolog considered was 5, and the largest possible homolog was included. Quality filters such as a minimum of coverage of 20, and a variant allele frequency (VAF) >5% was also implemented. The deletions which passed the above criteria were considered candidate MMEJ-dels.

### Identifying recurrent somatic and germline MMEJ-dels using the variant callers

The deletions containing microsatellites were initially filtered using HOMER (4.9.1) annotation of simple repeats, with a maximum length of three base pairs. The remaining mutations were divided as somatic deletions or germline deletions. Somatic deletions were defined as those with a VAF difference between the tumor and normal samples >6% and a population allele frequency less than 0.001. In addition, to be classified as somatic, at least 60% of the samples containing the MMEJ-dels at that genomic location were required to meet the aforementioned criteria. The remaining deletions were categorized as germline deletions. The recurrence of these somatic and germline MMEJ-dels was also accounted for.

### Identifying recurrent somatic and germline MMEJ-dels for Del-read algorithm

MMEJ-dels in the exome were identified using a list of all possible homologies with a size greater than or equal to five. To find homologies, exome sequences for hg19/hg18 were downloaded from the UCSC genome browser, and the MATLAB command ‘seqdotplot’ from the bioinformatics toolbox was modified to return the dot plot matrix without a visual output. The input parameters were 240 bp windows of the exome sequences, compared to itself, and the minimal size of homolog set to 5 bp with no mismatches allowed. The returned matrix contained homologies in desired size as true values. Consecutive and nested homologies were counted as the longest possible homolog for each segment. The starting coordinates of both homologies and the homolog length were recorded for downstream analysis.

The resulting homolog list was used to create specific ‘search sequences’ to identify reads containing MMEJ-dels. We focused our analysis on two kinds of MMEJ-dels, the perfect-match and the imperfect-match. In the perfect-match type, one of the homolog is retained, deleting the other homolog and the intervening bases. Whereas in the imperfect type, an extra base is deleted to the left (Type-A) or right (Type-B) of the perfect-match deletion.

To capture the deletion, a search sequence for each homolog was created by concatenating 15 bases upstream and downstream for each homolog pair using the Biopython's SeqIO module. The bam file was converted to Pysam alignment file. Then search sequence for each homolog pairs was searched in the aligned segment of the Pysam alignment, at a specific location using the fetch command. The specific location was all the reads mapping to 100 bases upstream and downstream to the homolog pair respectively. Finally, the total number of reads in which the search string is present was accounted. Depth was calculated using the count_coverage function of the class Pysam, by obtaining the mean coverage 5 bases upstream and downstream of the homolog pairs respectively.

### Filtering artifacts

Microsatellites were filtered using the Phobos program (version 3.3.12). MMEJ-del with microsatellite percent > 80 were removed. Additionally, to remove search-seq occurring in the reference genome, a fasta file of these sequences was created and then mapped to the reference genome using bwa. The resultant BAM was then filtered for complete matches using CIGAR field.

### Annotation

The MMEJ-dels were annotated using annovar (2017Jun01). All the variants were annotated with refGene, snp138NonFlagged, avsnp150, clinvar_20 190 305, cosmic70, 1000g2015aug_all, kaviar_20 150 923, abraom, ensGene, gme, icgc21, knownGene, nci60, gnomad211_genome databases. Further, Homer (4.9.1) was also used to obtain detailed annotations of the deleted and the surrounding genomic regions.

### *In silico* dataset

MMEJ deletions were simulated for this study. The reference genome (https://ftp-trace.ncbi.nlm.nih.gov/ReferenceSamples/giab/release/references/GRCh37/) and BAM file (ftp://ftp-trace.ncbi.nlm.nih.gov/ReferenceSamples/giab/data/ChineseTrio/HG005_NA24631_son/OsloUniversityHospital_Exome/151002_7001448_0359_AC7F6GANXX_Sample_HG005-EEogPU_v02-KIT-Av5_CGCATACA_L008.posiSrt.markDup.bam) were acquired from the Genomes in a Bottle database. The process involved selecting genomic coordinates in the bam file with coverage exceeding 100 using samtools depth, followed by the merging of these coordinates to create larger intervals. Subsequently, 5000 random MMEJ-dels were chosen, ensuring their placement within these intervals and preventing any overlaps between the deletions. This collection of deletions, along with the specified VAFs for each deletion, was used as input for the Bam surgeon Structural Variant script to generate a BAM file containing the simulated deletions and also requiring that the exact deletion is created, else discarded. This enabled us to construct a dataset of 617 MMEJ deletions. Following this, variant callers and the Del-read tool were implemented on the simulated bam file to determine their performance in identifying simulated MMEJ deletions.

### Defining candidate somatic and germline mutation using Del-read

Somatic deletions were defined with the criteria that VAF (tumor sample) >0.1 and VAF (control sample) = 0, with depth >30 in each sample. Additionally, it was required that the somatic mutation wasn’t found in the other datasets analyzed, for instance, BEAT- AML somatic deletions weren’t found in the BRCA dataset and *vice-versa*.

Germline deletions were defined with the criteria that both the paired tumor and the normal sample should have VAF between 0.1–0.86 and depth ≥30. Further targeted sequencing of healthy controls using Molecular Inversion Probes (MIPs) should validate their presence.

### Detecting minisatellites in MMEJ-dels

Minisatellites were detected using a tool called Tandem Repeat Finder/4.09 (18). For the input fasta file, 50 bases upstream to 50 bases downstream for each deletion were extracted. The tool was implemented with the standard parameters to obtain the length of repeats in each MMEJ-del. A list of minisatellites was obtained by filtering repeat lengths less than 5.

### Detecting G-quad in MMEJ-dels

For identification of the G-quad, the fasta sequence 20 bases upstream to 20 bases downstream of each MMEJ-del sequence was extracted. This was given as input to the Python implementation of G4 catchall tool (19). The input parameters were: allow tracts with two and three guanines, allow single imperfect tract, and allow search for an extreme loop up to size 30.

### Ribonucleoprotein (RNP) complex preparations

CRISPR/Cas9 experiments were done using sgRNAs guide (*ASXL1*) that were synthesized from IDT. Lyophilized sgRNAs were re-suspended in IDTE buffer (pH 7.5) to a final concentration of 100 uM. RNP complex for each reaction were generated by mixing 1.2 μl sgRNA, 1.7 μl Cas9 protein (10μg/10μl, IDT catalog number 1 000 735) and 2.1 μl PBS followed by incubation for 10 min at 20°C.

### Electroporation reactions

The electroporation reaction was performed using the 16-strip Lonza 4D nucleofector kit. Pre-electroporated cells were washed in PBS and spun down at 350×g for 10 min. 2 × 10**^5^** cells per reaction were re-suspended in 20 μl SF solution (catalog number PBC2-00675), a nucleofector, and added to the RNP complex or 2 μg px330 plasmid. FF-120 electroporation programs were used. Immediately after electroporation, pre-warmed media were added and cells were cultured at the same conditions as the pre-electroporation culturing for an additional 4 days before they were lysed for NGS sequencing.

### Cell lines pre-electroporation culturing

K562 cell line was used in this study. The cell line was obtained from ATCC. The cell line was sub-cultured two days before electroporation in RPMI 1640 Medium containing l-glutamine (Biological Industries, 01-100-1 A) with 10% FBS, streptomycin (20 mg/ml) and penicillin (20 unit/ml) at a density of 2 × 10^5^ cells/ml.

### K562 edited cell lines generation

K562 cells were electroporated using a sgRNA guide targeting the *ASXL1* gene, followed by a sorting for live single cells using BD FACSMelody™ Cell Sorter (BD Biosciences). Sorted cells were plated onto 96-well plates containing 120 μl/well RPMI 1640 Medium with L-Glutamine (Biological Industries, 01-100-1 A), 10% FBS, and penicillin (20 unit/ml). Seven days after sorting, 50 μl of fresh media were added to each well. Cells were further maintained by replacing 70 μl medium from each well once a week. Cell colonies were lysed 28 days after sorting for subsequent NGS sequencing. For cell lysis, cells were spun at 400×g for 10 min, cells pellets were mixed with 50 μl of 50 mM NaOH and heated at 99°C for 10 min. Then, the reactions were cooled down at room temperature and 5 μl 1 M Tris pH 8 was added to each reaction. NGS sequencing and analysis were performed. Colonies containing bi-allelic frameshift indels at the genomic loci of interest were further isolated and expanded.

### CRISPR/Cas9 experiments

20 bp sgRNA sequences were designed along the genomic loci of interest using ‘benchling’ and ‘deskgen’ algorithms. The first and second DSB at a specific position was performed using CRISPR/Cas9 using sgRNAs (*ASXL1*) that were synthesized from IDT (Supplementary Table S1).

### NGS library and targeted sequencing

For all NGS libraries, we used cell lysis products that served as a template for PCR amplification and library preparations. Dual indexed illumina Libraries were generated using two-step PCR procedure. 1st PCR primer prefix sequences and 2nd PCR primer sequences were used, similar to a previously described method. All relevant details are as follows: Target-specific primers were designed by Primer3plus (http://www.bioinformatics.nl/cgi-bin/primer3plus/primer3plus.cgi) and were ordered with the described 5′ prefixes (IDT) (Supplementary Table S2). 1st PCR was applied to target the regions of interest. The reaction mixture was composed of a PCR ready mix (using NEBNext® Ultra™ II Q5® Master Mix, NEB, M0544L), a cell lysis product and a final primer concentration of 1μM each. PCR protocol was as follows: 98°C for 30 s, followed by 40 amplification cycles of 98°C for 10 s, 65°C for 30 s and a final elongation at 65°C for 5 min. Following dilution of the 1st PCR products with nuclease free water (1:1000), a 2nd PCR was performed using primers composed of Illumina sequencing primers, indexes and adapters, under the same conditions as the 1st PCR with the exceptions of final primer concentration of 0.5μM each and 20 cycles of amplifications. Full sgRNA and primer sequences that were used throughout this study are provided in Supplementary Table S2. Barcoded 2nd PCR products were pooled together at equal volume. Pooled library sizes were selected (2% gel, BluePippin, Sage Science) and sent for 2 × 150-bp deep sequencing (Miniseq System, Illumina). The sequenced products were analysed by SIQ tool (20) and Del-read.

### MIP targeted sequencing probe design

MIP capture probes were designed using MIPgen (21) to capture an MMEJ mutation panel. Backbone and primer sequences were adopted from previous studies (22). MIPs were ordered either as single-strand MIPs (prepared as in Hiatt *et al.* (22)) or as an oligo mix (LCsciences, prepared as in Shen *et al.* (23))

### MIP capture protocol

One microliter DNA template was added to a hybridization mix together with a MIP pool (final concentration of 0.04 pM per probe) in 0.85 × Ampligase buffer. Mix was incubated in a thermal cycler at 98°C for 3 min, followed by 85°C for 30 min, 60°C for 60 min and 56°C for 60 min. Product was mixed with (final concentration in brackets): dNTPs (14 pM), Betaine (375 mM), NAD+ (1 mM), additional Ampligase buffer (0.5×), Ampligase (total of 1.25 U) and Q5 High-Fidelity DNA Polymerase (0.4 U, New England Biolabs). All the product of the hybridization was incubated at 56°C for 5 min followed by 72°C for 5 min. Enzymatic digestion of linear probes was performed by adding Exonuclease I (8U) and Exonuclease III (50 U). The mixture was incubated at 37°C for 10 min, followed by 80°C for 20 min. The final product was amplified using NEBNext Ultra II Q5 Master Mix (New England Biolabs). Samples were pooled and concentrated using AMPure XP beads at 0.75 × volumetric concentration and sequenced as the abovementioned described.

## Results

### Designing Del-read algorithm

**Figure 1.**
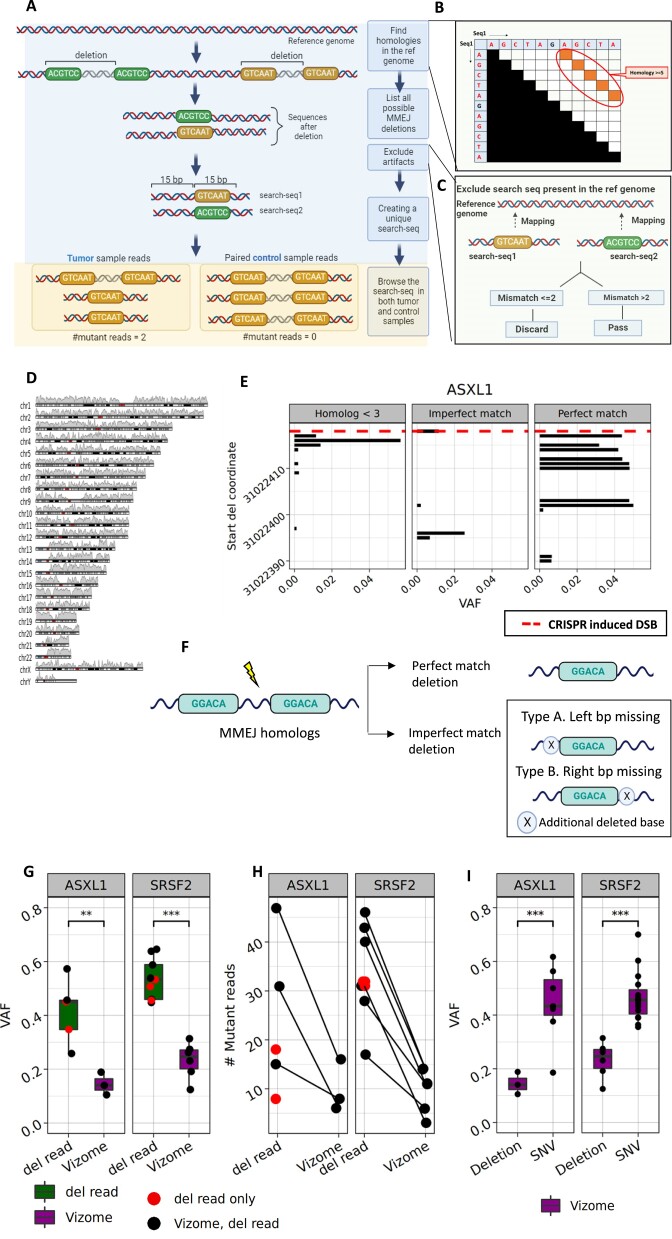
(**A**) Workflow for MMEJ-del detection in sequencing reads using homolog discovery. Colored rectangle blocks represent homologs. (**B**) Using matrices to discover homologs across exome, 240 bp at a time. (**C**) Filtering search sequences with a complete match in the reference genome. (**D**) Using karyoploteR to find the density of homologs across the exome (*N* ≥ 5). (**E**) VAF of mutations at different positions after induction of DSBs in *ASXL1* hotspot. (**F**) Summary representation of identified patterns of MMEJ-dels. Created with BioRender. (**G**) A box plot showing the VAF in patients with canonical *ASXL1* and *SRSF2* MMEJ deletions detected by Del-read (green) and Vizome (purple). P-values were calculated using a t-test unless stated otherwise (*P < 0.05, **P < 0.01, ***P < 0.001). Red dots indicate patients found exclusively by Del-read, and black dots indicate those found by both Del-read and Vizome. (**H**) A comparison between Del-read and Vizome showing the number of mutant reads detected in each patient with canonical *ASXL1* and *SRSF2* MMEJ deletions. Red dots indicate patients found exclusively by Del-read, and black dots indicate those found by both Del-read and Vizome. (**I**) Comparison of VAF of deletion and SNV as reported in Vizome.

We developed Del-read, an algorithm that leverages prior knowledge of DNA repair mechanism to detect all possible medium-sized MMEJ-dels. By locating homologies across the exome, a complete list of MMEJ-dels could be defined (Figure 1A). To discover the homologies, our strategy involved comparing the reference genome sequence to itself, 240 bases each time, resulting in a 240 × 240 matrix with a sliding window of 100 bases. A match along the diagonal in the upper triangle of the matrix indicated the presence of a homolog (Figure 1B). To make our algorithm computationally feasible, we restricted deletion size to less than 100 bp and a minimum homolog size ≥5. By iterating over all the chromosomes, we curated the first map of homologies across the exome (Figure 1D).

From each homolog, a corresponding sequence was created by concatenating 15 bases upstream and 15 downstream of the remaining homolog after the MMEJ-del. This sequence resembles an MMEJ-del event. But sequences, particularly those of low complexity (repetitive sequences), also resemble an MMEJ deletion event, causing false positive calls unrelated to MMEJ repair. Since MMEJ deletions in low complexity regions are difficult to discern from artifacts, we filtered them. Moreover, to eliminate these false positives, we mapped all the search sequences to the reference genome and eliminated complete matches (Figure 1C).

We then searched all the expected MMEJ-del events in sequencing reads from various datasets to identify both somatic and germline deletions. We assumed the presence of such a specific sequence (search-seq) in a read is highly indicative of an MMEJ-del. The count of detected MMEJ-del events was used to calculate the Variant Allele Frequency (VAF) of the deletion in that sample (Figure 1A).

### Patterns of MMEJ-dels

Until now, we assumed all MMEJ-dels arise from a perfect-match between the homologies. However, this assumption might be wrong and reduce the Del-read algorithm's sensitivity. To identify patterns of possible mismatches between the homologies, we reanalyzed previous *in vitro* CRISPR/Cas9 experiments (14). The experiments introduced specific DSB using CRISPR in the hotspot regions of *ASXL1* and *SRSF2* canonical MMEJ-dels. For this focused data, we created a set of search-seq corresponding to all possible deletions from size 6–100 bp. Our analysis identified both perfectly-matched homologies and novel imperfect-match homologies leading to MMEJ-dels, along with deletions with short homologies (*n* < 3) that could be potentially explained by NHEJ or POLQ mediated end joining (Figure 1E, Supplementary Figure S1A). The imperfect-match could further be broadly classified into two, where one additional base is missing from the left (Type A) or right (Type B) of the perfect-match deletion (Figure 1F). We discovered that imperfect-matched homologies were less common than perfectly-matched homologies (Figure 1E). This finding was also validated in the Catalogue Of Somatic Mutations In Cancer (COSMIC) dataset which revealed significant results (*P*< 0.001, chi-square test) (24) (Supplementary Figure S1B). Further inspection revealed the second most common *CALR* deletion (14) also followed the pattern of imperfect-match deletion (Supplementary Figure S1C). We provide examples of Type A and B imperfect-matches (Supplementary Figure S1D).

### Evaluation of the algorithm accuracy using the known MMEJ-dels

In the first step, we aimed to test whether our algorithm could detect the most common pre-leukemic MMEJ-dels in *ASXL1* and *SRSF2* in the Vizome database. Del-read not only detected these MMEJ deletions at higher VAFs in previously reported patients but also identified them in unreported patients (Figure 1G). Moreover, Del-read revealed a higher number of mutant reads that were previously missed in the data from reported patients, thus exhibiting an improved detection-rate of reads containing deletions (Figure 1H). To validate our results, we compared the performance of Del-read with other variant callers, specifically Platypus and Delly, which were not utilized for variant calling in the Vizome database. Remarkably, even these additional variant callers failed to detect all the deletions identified by Del-read (Supplementary Figure S1E) and they displayed diversity in VAF for deletion detection (Supplementary Figure S1F). Most variant callers generally show an underestimation of VAF for deletions compared to Single-Nucleotide Variants (SNVs) (25), as we also found in the Vizome database (Figure 1I). Del-read provided an expected VAF, similar to SNVs, for these deletions by detecting a higher number of mutant reads (Figure 1G, H). Altogether, Del-read captures mutations in individuals missed in the currently available databases, even for commonly known deletions, and further improves VAF estimate, which might be clinically important (26). Its adaptability extends to diverse organisms, demonstrated with the mouse genome mm10 (Supplementary Figure S1G), and to detect all the possible deletions in a subset of a region. Based on these findings, we next aimed to assess the sensitivity and specificity of Del-read in simulated data.

### Assessing Del-read accuracy in simulated data

**Figure 2.**
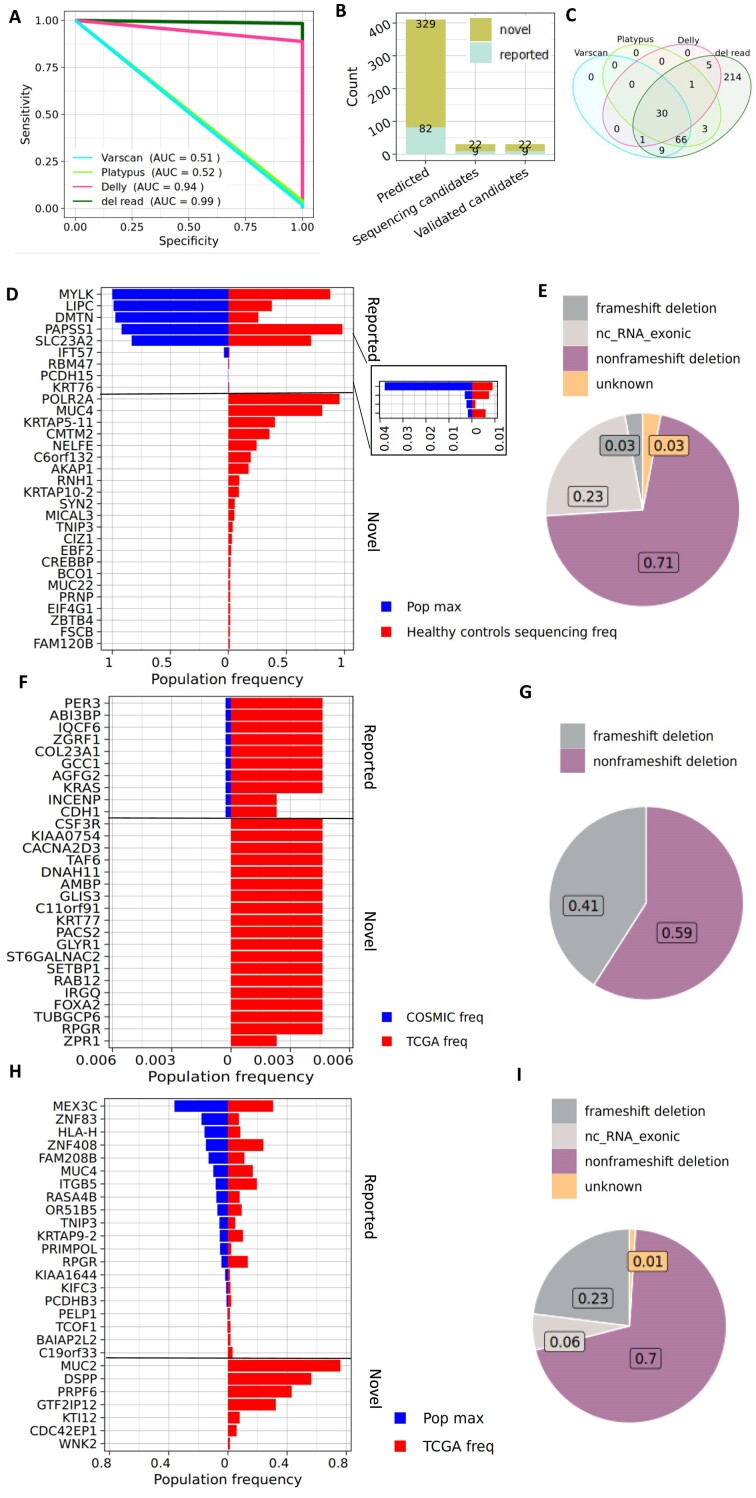
(**A**) Sensitivity versus specificity of calls on a BAM with simulated MMEJ deletions. (**B**) Bar plot of the number of novel and reported MMEJ-del detected across discovery (predicted) and validation. Sequencing candidates are MMEJ deletions selected for sequencing. Validated candidates are among the sequenced deletions found in patient samples. (**C**) Comparison of novel MMEJ deletions called by Del-read to other variant callers (**D**) Comparison of population frequency of recurrently mutated germline MMEJ-del from BEAT-AML found by targeted sequencing validation in ethnicity matched healthy controls (red), and in 1000 genomes dataset (blue). (**F**) Comparison of population frequency of somatic candidates in the TCGA-BRCA (red) and the COSMIC (blue) datasets (**H**) Comparison of population frequency of germline candidates (Frequency > 0.01) in the TCGA-BRCA (red) and 1000 genomes (blue) dataset. (**E**, **G**, **I**) Pie chart distributions of functional consequences of MMEJ-del from (**E**) targeted sequencing of germline candidates found in BEAT-AML (**G**), somatic candidates found in TCGA BRCA, (**I**) and germline candidates found in TCGA-BRCA.

We next set out to determine the overall performance of our tools by creating artificial MMEJ-dels on top of real data obtained from the Genomes In A Bottle (GIAB) database. We simulated 617 medium-sized deletions to obtain the sensitivity and specificity of Del-read in comparison to other variant-callers. Del-read had the highest sensitivity (98.3%) and the highest specificity (100%), followed by Delly (sensitivity = 88.7%, specificity = 100%) (Figure 2A). Once we established confidence in our calls based on simulated data, we aimed to identify novel somatic and germline MMEJ-dels.

### The identification of novel somatic and germline MMEJ-dels

First, we applied Del-read to a publicly available dataset of 359 paired (tumor-normal tissue) BEAT-AML samples and to 224 paired and additionally 200 unpaired TCGA Breast cancer samples. The deletions found were further categorized into somatic or germline deletions (Supplementary Figure S2A, Methods). In the BEAT-AML dataset, we didn’t identify any novel somatic deletions but identified 329 novel germline MMEJ-dels and 82 reported MMEJ-dels (Figure 2B).

The novel MMEJ-dels distribute along the chromosome, as shown in Supplementary Figure S2B, and their deletion length, as in Supplementary Figure S2C. Similarly, applying other variant callers on the BEAT-AML dataset did not identify novel somatic deletions. Moreover, the variant callers showed low overlap (∼10%) in calling germline MMEJ-dels (Supplementary Figure S2D). We noticed that Delly could identify mainly larger deletions while Varscan and Platypus identified shorter deletions (Supplementary Figure S2E). Accordingly, we tested whether, by combining Varscan, Platypus, and Delly one could identify all the novel germline deletions found by Del-read. However, we found that 65% of the deletions found by Del-read were not called by any of the other callers (Figure 2C). We chose 31 (22 novel deletions and 9 reported) to validate by targeted MIP-based sequencing in a cohort of 672 ethnicity-matched healthy controls (Figure 2B). All the reported germline MMEJ-del, classified into common and rare based on their frequency in the population, were detected at comparable frequencies to those found in the gnomAD database (Figure 2D). Moreover, we were able to identify all 31 deletions in this validation cohort. This suggests that there are hundreds of undescribed germline MMEJ-dels in the exome and much more than that in the human genome. Most of the novel germline deletions identified by Del-read were non-frameshift deletions (Figure 2E).

In addition, we applied the Del-read algorithm to TCGA breast cancer samples. We discovered 29 somatic and 96 germline MMEJ-dels. Among the somatic deletions, ten were previously reported in the COSMIC database, including deletions in *KRAS* and *CDH1*, and 19 were novel (Figure 2F). The proportion of frameshift MMEJ-dels was higher in somatic deletions (Figure 2G). Interestingly, we observed that the mean length of the novel somatic deletions was significantly longer (=45) than of the reported mutations (=19) (Supplementary Figure S2F) suggesting that our algorithm is helpful in identifying clinically relevant cryptic medium-sized deletions that were so far missed by other methods. Apart from the somatic deletions, we also discovered 35 novel and 61 reported germline deletions of which 31 appeared with population frequency >0.01 (Figure 2H) enriched in non-frameshift deletions (Figure 2I).

### Enrichment of minisatellite repeats and G-quadruplex motifs in MMEJ-dels

**Figure 3.**
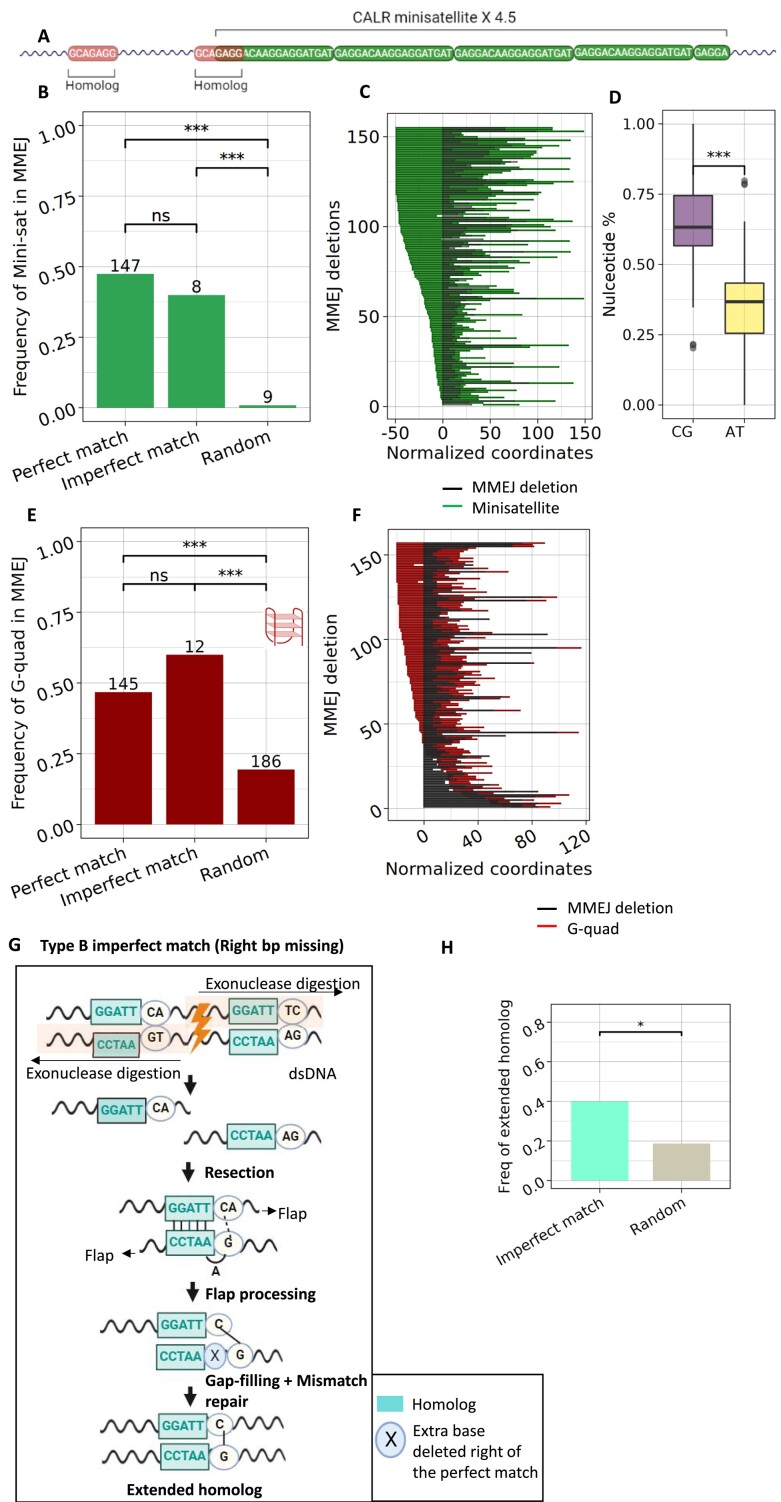
(**A**) Schematic of *CALR* homolog (red) and the adjacent minisatellite (green) repeated 4.5 times. (**B**) Barplot of enrichment of minisatellites in categories of MMEJ-del from BEAT-AML. The values above bars indicate the number of deletions that have minisatellites in that category. P values were calculated by two-proportion z-test with Bonferroni correction unless indicated otherwise *P < 0.05, **P < 0.01, ***P< 0.001. (**C**) Relative position of each minisatellite (black) w.r.t. the corresponding MMEJ-del (red). (**D**) Boxplot of nucleotides enrichment in minisatellite. P values were calculated by Student's *t*-test. *P < 0.05, **P < 0.01, ***P< 0.001. (**E**) Barplot of enrichment of G-quadruplex in categories of MMEJ-del. The values above bars indicate the number of deletions that have G-quadruplex in that category (**F**) Relative position of each G-quadruplex (red) w.r.t. the corresponding MMEJ-del (black). (**G**) Putative mechanism of type B imperfect-match MMEJ-del (One-bp extra missing to the right of the perfect-match deletion) formation after the DSBs. Created with BioRender. (**H**) Proportion of imperfect-match deletions with an extended homolog compared to random MMEJ-del.

It is known that specific local sequence contexts may be prone to double-stranded DNA breaks (27), thus elevating the likelihood of generating MMEJ. The magnitude of the discovery of novel MMEJ-dels improved our power to detect cryptic genomic features, which might be correlated with the presence of MMEJ-dels. Previous studies in yeast have suggested that tandem sequence repeats of >5 bases, i.e. minisatellites have the potential to induce stalled replication forks, resulting in DSBs and gross chromosomal rearrangements (28). This is probably due to their ability to form secondary structures which impede DNA replication. However, the association between minisatellites and MMEJ-dels has not been investigated. Here, we observed for the first time a tandem sequence repeats of 18 bases repeating 4.5 times overlapping with the most common MMEJ-del in myeloid malignancy, *CALR* (Figure 3A). Subsequently, we evaluated the role of minisatellites in other MMEJ-dels by analyzing two datasets- BEAT-AML and COSMIC (Figure 3B, Supplementary Figure S3A respectively). The datasets demonstrated that MMEJ-dels were significantly enriched in regions with minisatellites compared to non-MMEJ-dels and random deletions (*P* < 0.0001, with Bonferroni correction). Also, the imperfect-match deletions showed comparable enrichment of minisatellites to perfect-match deletions. Next, comparing their relative position with the MMEJ-del coordinates normalized to zero showed that in more than 99% of the cases, the minisatellite overlapped with the deletion, as opposed to flanking on either side (Figure 3C, Supplementary Figure S3B). Moreover, sequence enrichment analysis revealed the GC-rich nature of minisatellites (Figure 3D, Supplementary Figure S3C), implying their potential to form G-quadruplex (G-quad) structures during replication (29,30). G-quad motifs are known to cluster at micro (mini)satellite repeats and crucial regulatory elements like promoters (31). We used the G4 catchall tool (19) to assess whether MMEJ-dels are G-quad-prone genomic regions. We found that in both the BEAT-AML and COMIC dataset (Figure 3E, Supplementary Figure S3D, respectively), MMEJ-dels were significantly enriched in G-quads compared to the random sequences (*P* < 0.0001). However, G-quadruplexes were not enriched at GC-rich minisatellites suggesting there are two independent routes to MMEJ deletion formation. Similar to minisatellites, G-quads also did not show bias towards the left or right side of the MMEJ-del (Figure 3F, Supplementary Figure S3E, respectively).

### Mechanism of the novel pattern of MMEJ-dels

To summarize this part, we observed enrichment of MMEJ-dels in minisatellite repeats and in regions predicted to form G-quadruplexes which could at least in part explain why MMEJ-dels are created during replication (14). We could not explain why imperfectly-matched homologies were created in some cases. Such imperfectly-match deletions occur at a lower frequency, about 10% of the perfect-match deletions (Supplementary Figure S1B). Initially, it was speculated that the occurrence of imperfectly-matched deletions might be attributed to the involvement of shorter homologs, but delineating the mechanism (Supplementary Figure S3F) suggested otherwise. We then considered that this can occur due to mispairing during MMEJ repair. Moreover, in our previous report, we observed that DSBs within the homologies lead to fewer MMEJ-dels compared to DSB in between the homologies (14) implying longer homologies are more stable than shorter ones. Altogether, imperfectly-matched deletions could occur because the MMEJ repair mechanism attempts to create a larger homology at the expense of mispairing during the homology alignment. Illustrated graphically (Figure 3G), during MMEJ repair following resection, the potential for forming a larger homolog arises through base pairing between C–G on the right side of the paired homologies. Consequently, the unpaired base ‘A’ is excised through base excision repair, resulting in an additional one bp deletion on the right side of the homolog. To test this hypothesis, we compared the fraction of imperfect-deletions with the potential to form a larger homolog to the fraction that cannot. We found a significant proportion of imperfect-deletions with larger homolog potential in BEAT-AML (Figure 3H).

### Effect of editing the canonical *ASXL1* homolog on MMEJ repair

**Figure 4.**
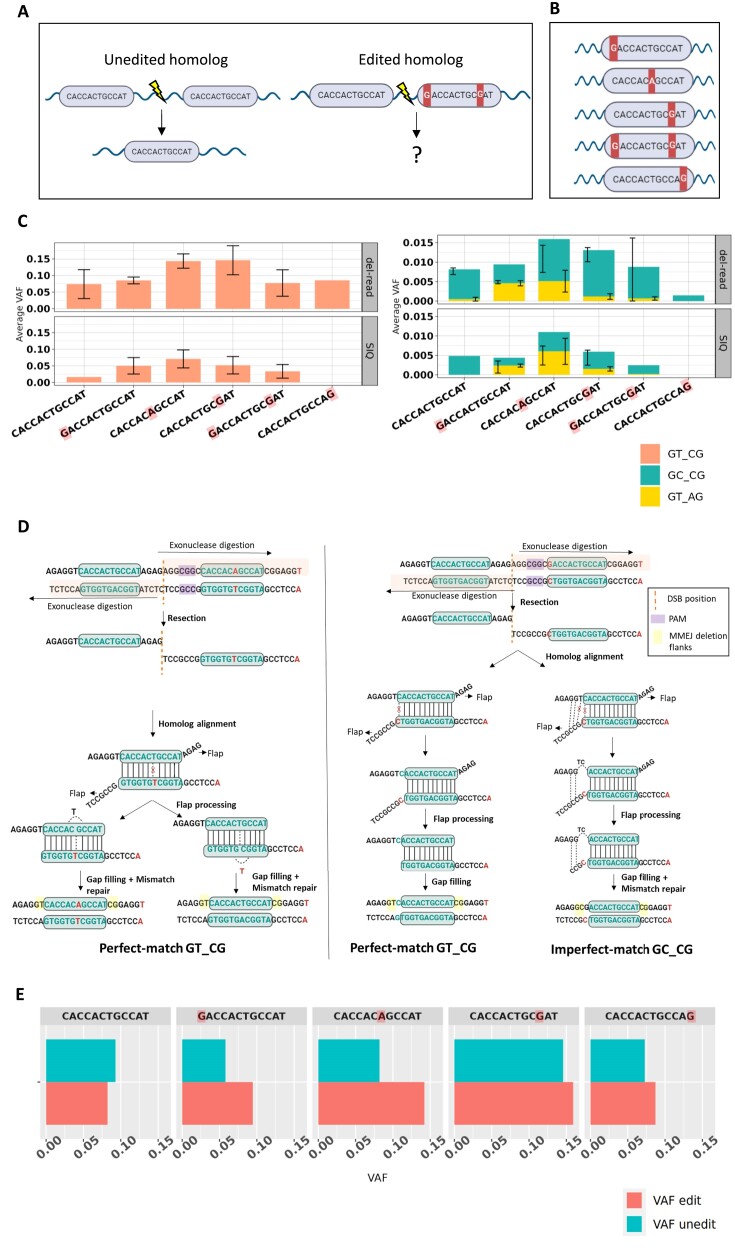
(**A**) Schematic of the effect of homolog change on the formation of MMEJ-del. Created with BioRender. (**B**) Introduction of different edits in the second *ASXL1* homolog using CRISPR at the described location. Created with BioRender. (**C**) Comparison of Del-read and SIQ for perfect-match and diverse imperfect-match deletions. (**D**) Left panel illustrates the mechanism of a perfect-match deletion when an edit occurs between the homologs, resulting in ‘GT_CG’ as the flanks. The highlighted areas in yellow represent the flanks. Middle panel demonstrates the mechanism of a perfect-match deletion with a mismatch at the first base of the homolog, leading to ‘GT_CG’ as the flanks. Right Panel: Depicts the mechanism of an imperfect-match deletion where the homolog is extended to the left due to mispairing, resulting in ‘GC_CG’ as the flanks. Created with BioRender. (**E**) Effect of homology edits on canonical *ASXL1* achieved through another CRISPR-mediated double-strand breaks in the *ASXL1* gene. This experiment was performed on K562 cell line. X-axis represents the VAF of mutations, and Y-axis represents the mutation change.

MMEJ repair involves the base pairing between the homologies not subjected to exo-nucleolysis. It implies that introducing mismatches in the homolog, and creating mispairing similar to imperfect-matches potentially affect this pairing process. To explore this possibility, we aimed to introduce mismatches by editing different nucleotides along the homolog arm of the *ASXL1* canonical MMEJ-del. This was done by modifying one of the homologies using CRISPR/Cas9 and different single-stranded oligodeoxynucleotide (ssODN) donors in K562 cells (Figure 4A, B). First, we performed the CRISPR Homologous directed repair (HDR) to edit the cells followed by single-cell sorting and then sequencing the single-cell colonies to identify colonies with particular mutations in the homolog arm. In the next step, we planned to introduce DSB between the two homologies by a different CRISPR guide to recapitulate the canonical MMEJ-del. However, we specifically chose colonies in which the heterozygous mutations were introduced, except for two mismatch introductions, so that we could observe the effect of DSB on both the edited and unedited alleles (as an internal control). Our observations revealed that editing had no impact on the repair outcome compared to the unedited state (Supplementary Figure S4A, dotted box). Additionally, edits at different positions still led to the *ASXL1* deletion formation and did not have any significant effect on the mutations in the vicinity as detailed in Supplementary Figure S4A and Supplementary Figure S4B, suggesting MMEJ is robust towards mismatches in the homolog. But a deeper exploration into the canonical *ASXL1* deletion with introduced mismatches using SIQ (20) (a tool to analyse CRISPR data), and Del-read yielded compelling findings.

Intriguingly, we observed a diverse array of imperfect-matches, reinforcing the notion that the MMEJ repair mechanism tends to generate larger homologies due to mispairing. As described earlier, mispairing can cause a base to lack a partner, leading to an extra one-base deletion alongside the MMEJ deletion. We classified these as Type A or Type B imperfect-match deletions, depending on whether the deleted base is positioned to the left or right of the retained homolog sequence. Mispairing can also lead to repairs where no deletion occurs, but due to a mismatch, bases are removed from one strand and correctly paired with complementary bases. This results in other types of imperfect-match deletions. When there is no mispairing at the flanks, like the perfect-match deletion, canonical *ASXL1* deletions occur at the highest frequency (Figure 4C, left panel). This is represented by ‘GT_CG’ in Figure 4C, where ‘GT’ is the flank on the left, and ‘CG’ is the flank on the right after the perfect-match *ASXL1* deletion. However, when the mechanism attempts to create a larger homolog, with the consequence of mismatches, the deletion frequency was notably reduced, mirroring a tenfold decrease (‘GC_CG’ and ‘GT_AG’), similar to imperfect-match Type A and Type B scenarios (Figure 4C, right panel). ‘GT_CG’ can be explained by the perfect pairing of the flanks post homolog alignment (Figure 4D left and middle panels). In this context, we depict two scenarios: an edit occurring between homologs and an edit at the homolog's first base. Notably, an edit in between yields two possibilities in MMEJ repair—retaining or omitting the edit. Conversely, an edit at the first base results in MMEJ outcome without preserving the edit. The flank ‘GC_CG’ can be explained by the mechanism using the left flap and leveraging the availability of two additional G–C pairings to extend the homolog (Figure 4D, rightmost panel). This process is succeeded by base excision of the unpaired bases ‘TC’, along with the conventional MMEJ steps of flap processing and gap filling. Similarly, GT_AG can be explained by the mechanism using the right flap to extend the homolog leveraging an extra G–C pairing present on the right of the paired homolog, followed by the execution of similar MMEJ steps (Supplementary Figure S5A, left panel). Simultaneous efforts may occur to extend homologs on both sides that may lead to ‘GC_AG’ at the flanks of the retained homolog. The data suggests such mechanism is less likely and may be explained by a dramatic hundred-fold decrease in deletion frequency which places it in the order of magnitude of the amplicon sequencing error. Hence, it is difficult to determine their presence, and therefore, we report only the putative mechanism of such deletions. In order to show that the imperfect-match deletions are not caused due to sequencing error we reanalyzed the experiments without the CRISPR/Cas9 DSB (ASXL1_noRNP) (14), and found very few imperfect-match deletions, if any, comparing it with breaks at different positions in canonical *ASXL1* (Supplementary Figure S5B, Supplementary Table S3). Finally, combining perfect and imperfect-match *ASXL1* deletions across various edits and comparing them with the unedited allele reveals no discernible effect of the edit on the repair process. (Figure 4E).

In comparing our tool Del-read with the CRISPR-specific tool SIQ, we revealed our ability to identify deletions missed by the SIQ tool (for ex., Mismatch position 0 (GT_AG), Mismatch position 12 (GC_CG)), and we demonstrated higher sensitivity in detecting deletions at an elevated VAF. When analyzing CRISPR experiments we propose a two-step approach: first, utilizing the SIQ tool to identify all possible deletions and then employing our tool to refine the VAF estimates and discover additional deletions based on the preexisting knowledge of the underlying mechanism.

## Discussion

Collectively, our findings provide novel methodology for the detection of MMEJ-dels and hotspot deletions (Figure 1). We found in published datasets hundreds of novel germline and somatic MMEJ-dels and validated some of the germline deletions by targeted sequencing of healthy individuals (*N* = 672) (Figure 2). The large number (*N* = 329) of novel discovered MMEJ-dels from the BEAT-AML dataset enabled us to better describe genomic features enriched in the sequence around MMEJ-dels (Minisatellites and G-quadruplexes) (Figure 3). Interestingly, we also observed a new pattern of MMEJ-dels, characterized by imperfect-matches between homologies. We tested the impact of mismatches on the repair process and found no significant difference in the outcomes between the edited and unedited alleles, challenging assumptions about the potential impact of mismatches. The observed patterns of decreased deletion frequency in extended homolog scenarios provide insights for refining genome editing strategies, offering potential applications in precise therapeutic interventions

The discovery of medium-size deletions remains an outstanding challenge. Most available data comes from short-read sequencing that has not been adequately analyzed. While long-read sequencing may provide some resolution to these challenges, such data is yet to be generated. Our a priori annotation of all homologies in the exome allowed us to identify MMEJ-dels better. Similarly, the knowledge of *FLT3* internal non-frameshift tandem duplication (FLT3/ITD) can be used to identify the hard-to-detect duplications in *FLT3*. Our method Del-read is still computationally heavy (0.004 s/deletion), and improvements in it will allow us to scan all available datasets and whole genomes.

The magnitude of MMEJ-del identified in our study allowed us to explore the possible mechanisms underlying their formation. Notably, we noticed a unique pattern of tandem repeat (of 18 nucleotides) in *CALR* gene that started within the second homolog and continued 4.5 times. This finding led us to discover that MMEJ-dels are enriched in GC-rich minisatellites and G-quadruplexes. While we do not fully understand the mechanisms behind such associations, it hypothesizes that secondary structures, which are known facilitators of DSB, could be involved in creating MMEJ-dels. More mechanistic studies on the role of minisatellites and G-quadruplexes in MMEJ-del formation are needed.

In addition to our Del-read method, we employed an unbiased approach to detect all possible deletions within a small genomic region. By introducing a double-strand break and utilizing search-seq designed to capture every possible deletion length (up to 100 bp), we identified imperfect-matched MMEJ deletions that were not captured by other variant callers. In particular, we discovered MMEJ-dels with a single base pair missing from the left or right side of the retained homolog. Although less prevalent, indicating a lower tolerance for mismatches in the homologies, these imperfectly-matched MMEJ-dels were identified in available sequencing datasets. These deletions were supported by the extended homolog hypothesis but we cannot rule out the possibility of the contribution of error-prone polymerase to the process (11). Since homology pairing between the two DNA strands is a crucial step for the repair, we hypothesized that this could be affected by introducing mismatches in the homologies. To prove this hypothesis, we designed genome editing experiments in which we created mismatches between homologies by introducing mutations in different positions along the homolog arm. The observed lack of significant differences between edited and unedited alleles in MMEJ repair suggests a robustness in the repair system, highlighting its reliability and potential for therapeutic applications. However, investigating the canonical *ASXL1* deletion with mismatches uncovered diverse imperfect-matches, emphasizing MMEJ’s tendency to accommodate mispairing and potentially extend homologies.

Altogether, our study contributes tools and insights into the detection, characterization, and enhances our understanding of the intricacies of MMEJ repair mechanisms, laying the foundation for future research and therapeutic advancements.

## Supplementary Material

gkae1132_Supplemental_Files

## Data Availability

The healthy controls data can be accessed at the accession number E-MTAB-13306: https://www.ebi.ac.uk/biostudies/arrayexpress/studies/E-MTAB-13306. The code for Del-read and analysis is available at https://github.com/LiranShlush/MMEJ. Human homologies are available at: https://www.dropbox.com/scl/fo/6kxlg4fvbgwyg00y3hi53/h?rlkey=77slhi1ycjxnhpz1oid7hciuk&dl=0. Mouse homologies are available at: https://github.com/LiranShlush/MMEJ_homologies. CRISPR data is available at: https://www.dropbox.com/scl/fo/u6vnhuqe9rk309ip8jmd0/h?rlkey=zaagqr4owi66kpj9340shkwlp&dl=0. Datasets have been archived in Zenodo: https://doi.org/10.5281/zenodo.13996762. Code and analysis have been archived in Zenodo: https://doi.org/10.5281/zenodo.13996734.
