# Supplementary material for: Utilizing insights of DNA repair machinery to discover MMEJ deletions and novel mechanisms
Source: Nucleic Acids Res. 2024 Nov 28;52(22):e106. doi: 10.1093/nar/gkae1132 (PMC11662932; doi:10.1093/nar/gkae1132)
Supplement: gkae1132_Supplemental_Files [file gkae1132_supplemental_files.zip › 3_10_24_extended_figures.pdf]

Supplementary Figure S1

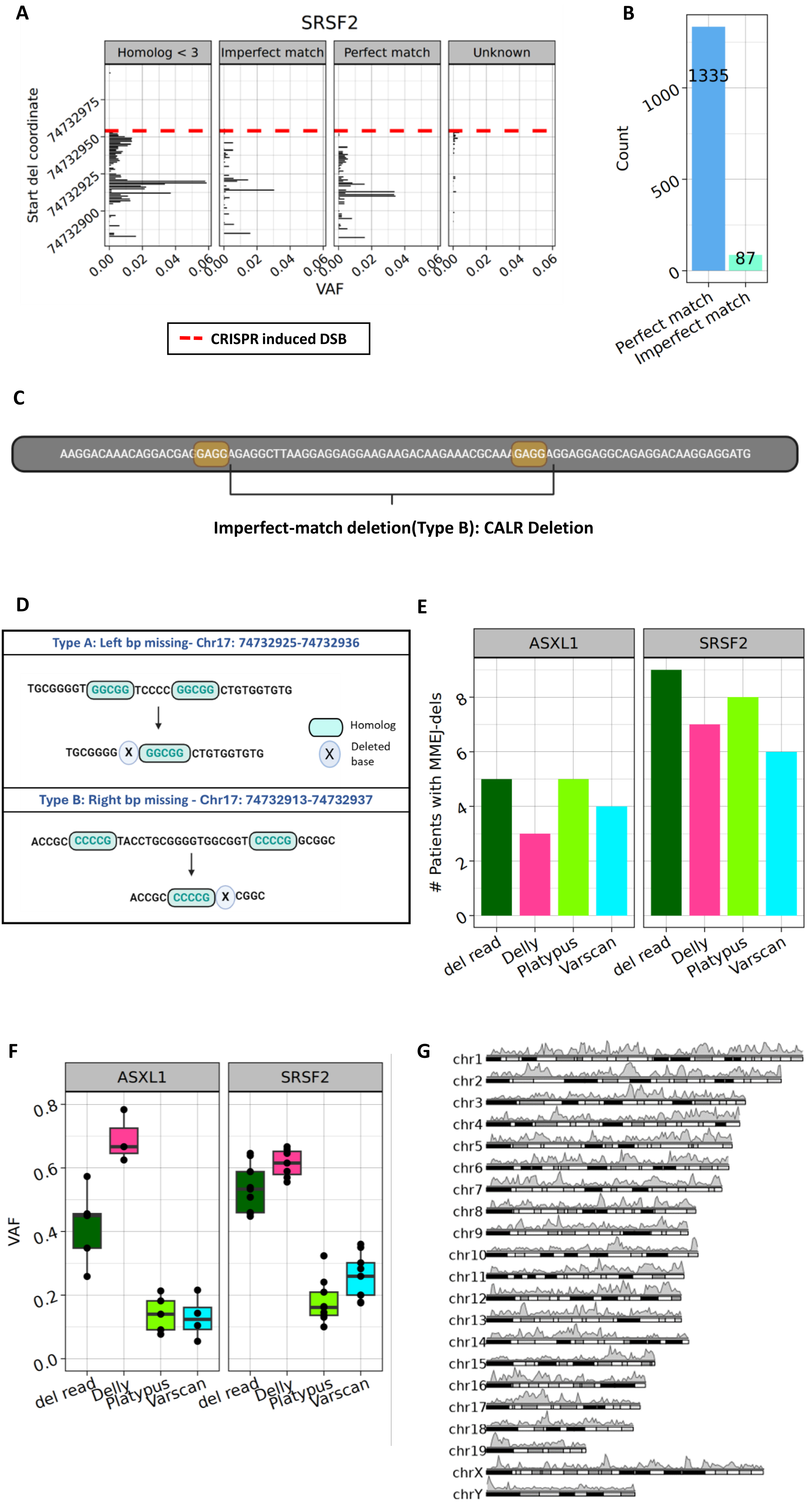

Supplementary Figure S1(A) VAF of mutations at different positions after induction of DSBs in the *SRSF2* hotspot. **(B)** Number of perfect match and imperfect match deletions in the COSMIC database. **(C)** Imperfect MMEJ deletion type-B in *CALR*. The deletion is marked in brackets and the potential homologs are marked in yellow. **(D)** Type A and B imperfect matches examples **(E)** Number of patients with the canonical *ASXL1* and *SRSF2* deletion as detected by four algorithms in the BEAT AML database and **(F)** A box plot showing VAF of the canonical *ASXL1* and *SRSF2* deletions as detected by the algorithms applied on the BEAT AML database **(G)** Using karyoploteR to find the density of homologs(N ≥ 5) across the exome in mice mm10.

Supplementary Figure S2

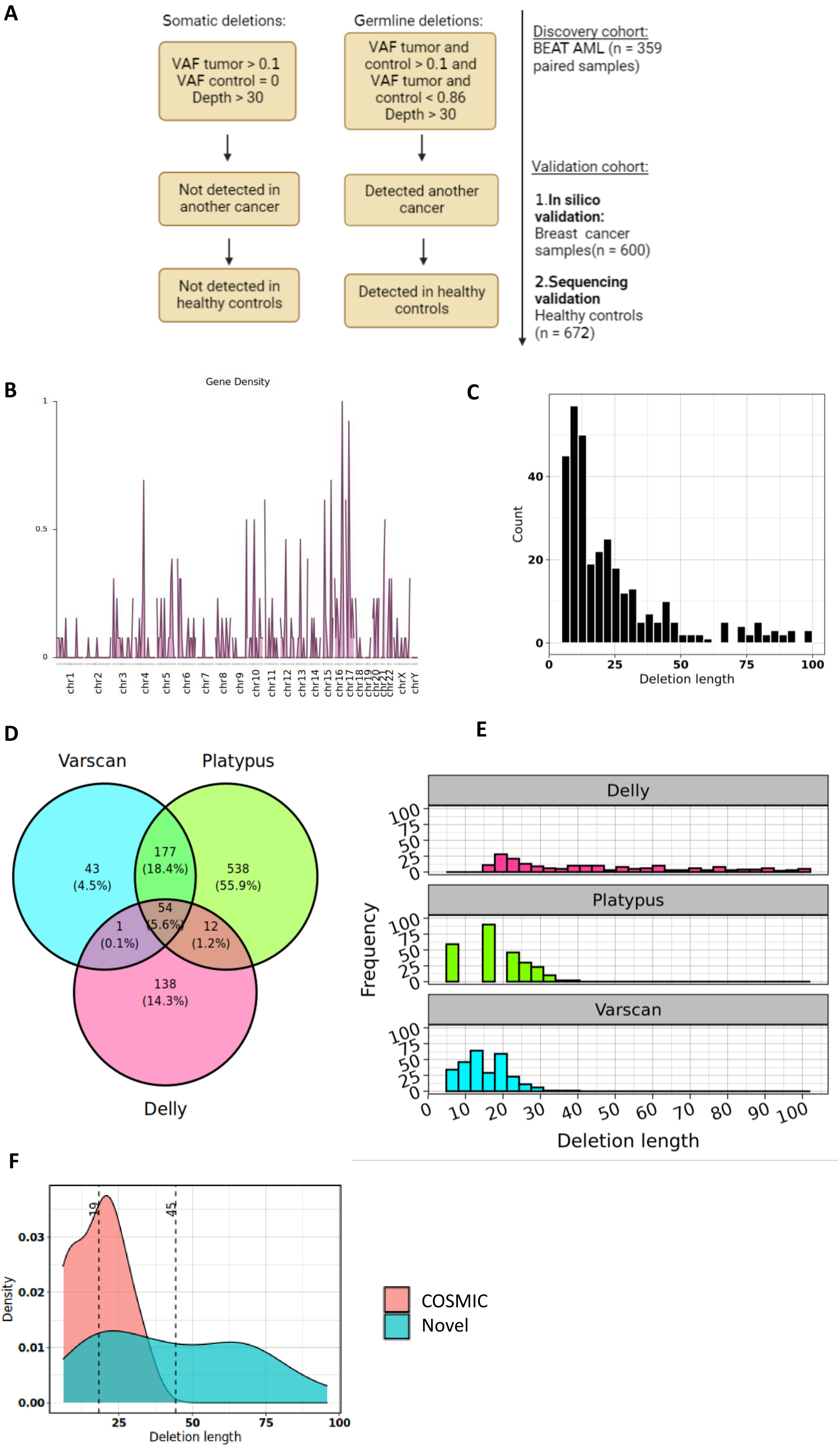

Supplementary Figure S2(A) Flowchart for categorizing MMEJ-del as somatic or germline. **(B)** Distribution of predicted germline MMEJ-del candidates across chromosomes in BEAT AML. **(C)** Histogram of deletion length for the predicted MMEJ-del in BEAT AML **(D)** Venn diagram of MMEJ-del calls by each variant caller. **(E)** Histogram distribution of deletion length by variant callers. **(F)** Density distribution of novel and reported somatic MMEJ-del length in TCGA-BRCA.

Supplementary Figure S3

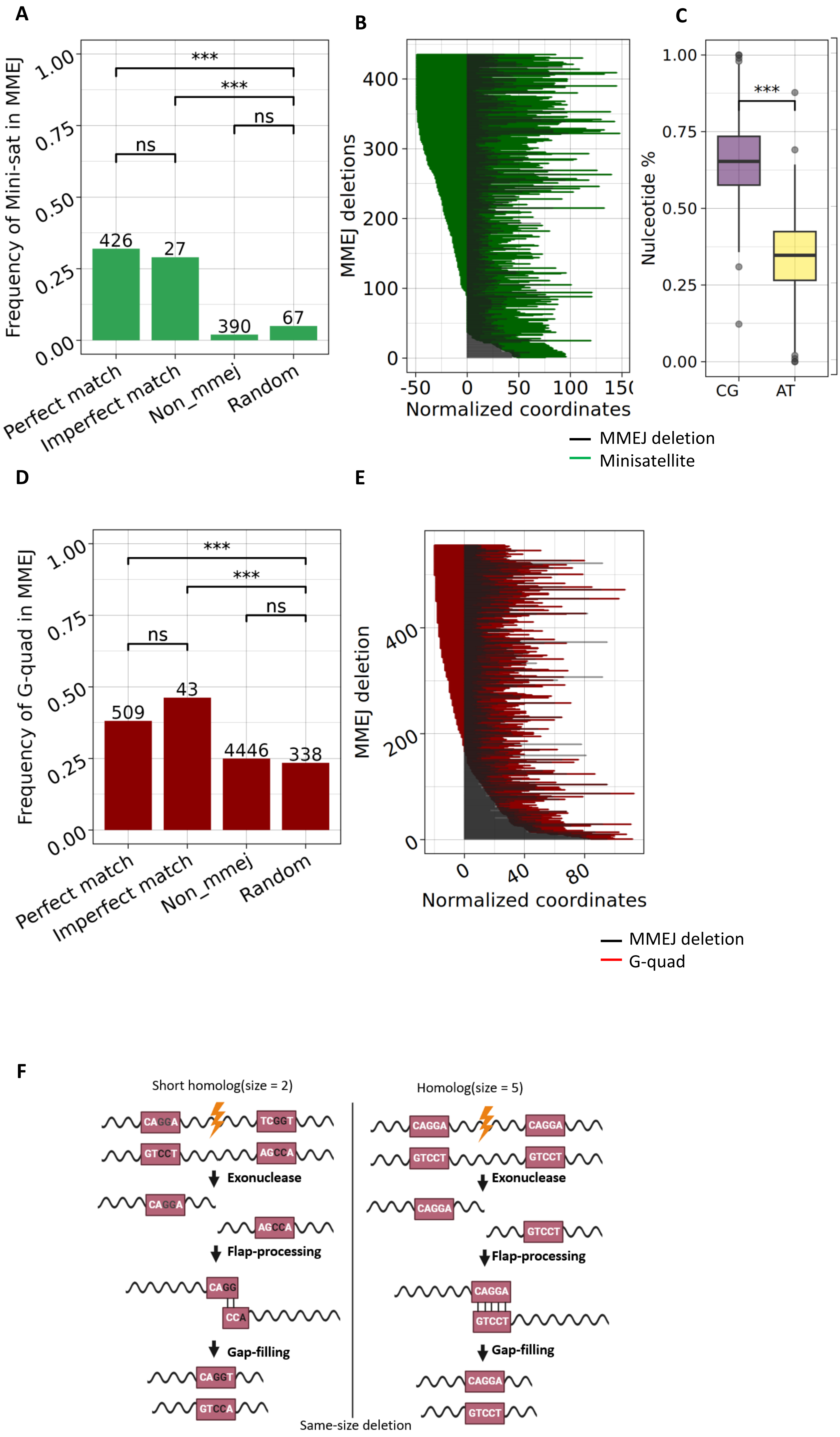

Supplementary Figure S3(A) Barplot of enrichment of minisatellites in categories of MMEJ-del from the COSMIC database. The values above bars indicate the number of deletions that have minisatellites in that category. P values were calculated by two-proportion z-test with Bonferroni correction unless indicated otherwise. *P < 0.05, **P < 0.01, ***P<0.001 **(B)** Relative position of minisatellite (green) w.r.t. the MMEJ-del (black). **(C)** Boxplot of nucleotide enrichment in minisatellite. P values were calculated by student's t-test. *P < 0.05, **P < 0.01, ***P<0.001 **(D)** Barplot of enrichment of G-quadruplex in categories of MMEJ-del from the COSMIC database. The values above bars indicate the number of deletions that have minisatellites in that category **(E)** Relative position of G-quadruplex (red) w.r.t MMEJ deletion (black). **(F)** Proposed mechanism illustrating that homolog length does not affect the MMEJ deletion size.

Supplementary Figure S4

A

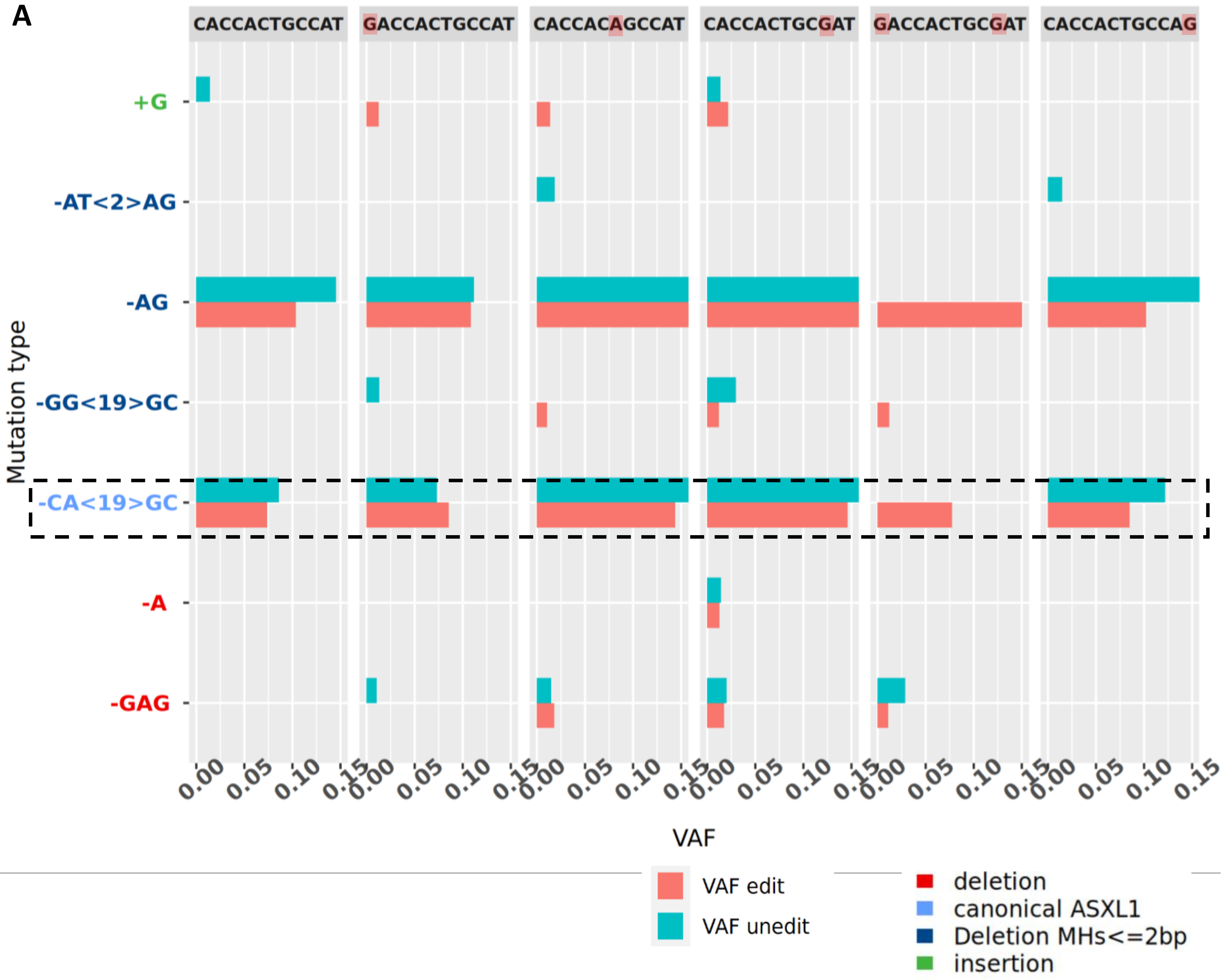

B

mutations	types	Start	End
-A	deletion	31022419	31022419
-GAG	deletion	31022416	31022418
-CA<19>GC	canonical ASXL1	31022403	31022425
-AG	Deletion MHs<=2bp	31022415	31022416
-GG<19>GC	Deletion MHs<=2bp	31022400	31022422
-AT<2>AG	Deletion MHs<=2bp	31022413	31022418
+G	insertion	31022417	31022417

Supplementary Figure S4(A) Effect of homology edits on diverse mutations achieved through another CRISPR-mediated double-strand breaks in the *ASXL1* gene. This experiment was performed on K562 cell line. X-axis represents the VAF of mutations, and Y-axis represents the mutation change. The '+' sign before a mutation change indicates an insertion and '-' indicates a deletion). Colors on the Y-axis show the categories of mutation (deletion, canonical *ASXL1*, deletions with MH, and insertion, as described in the legend). The dashed box represents the perfect-match canonical *ASXL1* deletion. Depicted here VAF > 0.01 **(B)** Description of the mutations in the Supplementary Figure S4A – the start position, the end position and the sequence that was deleted or inserted.

Supplementary Figure S5

A

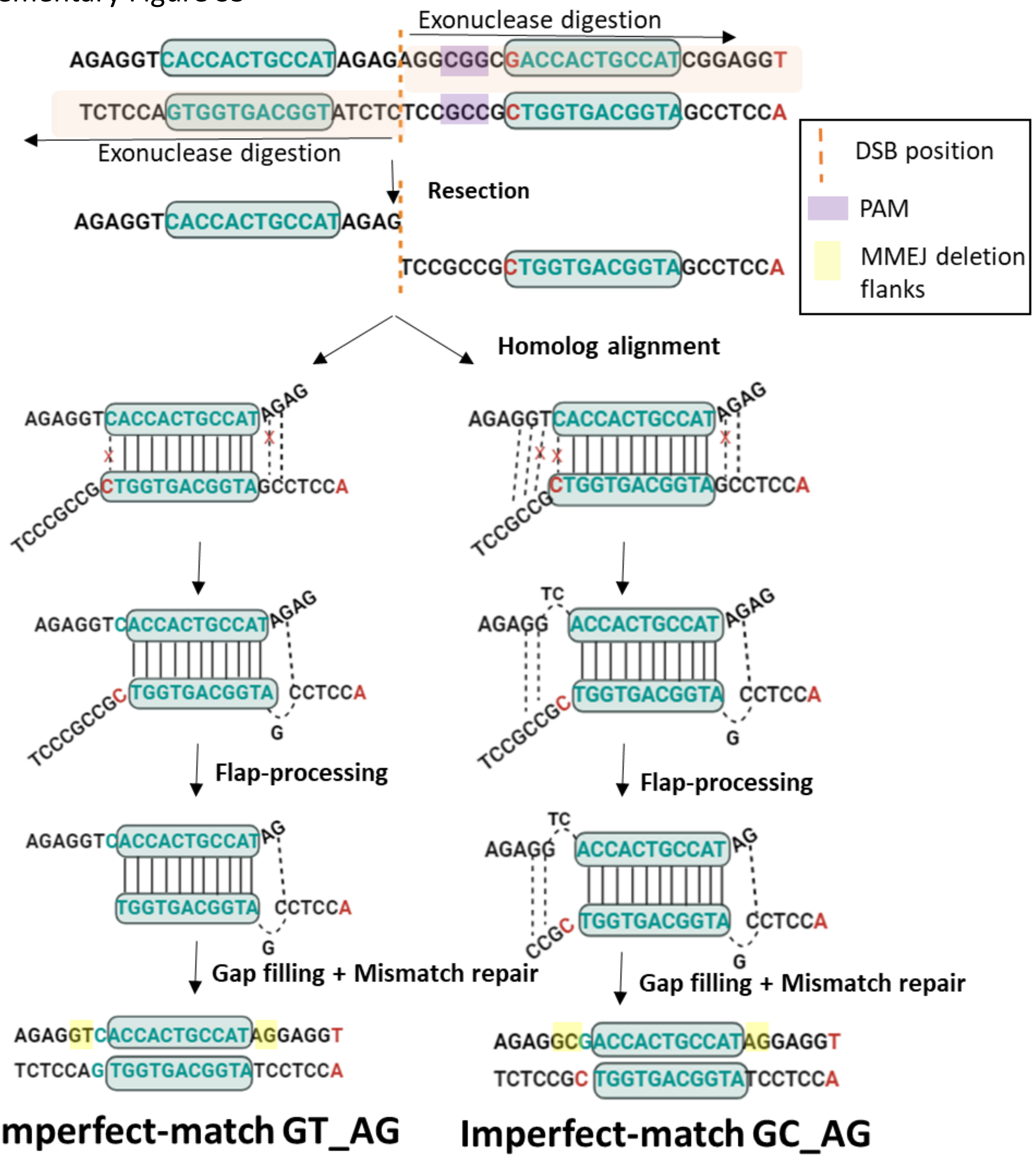

B

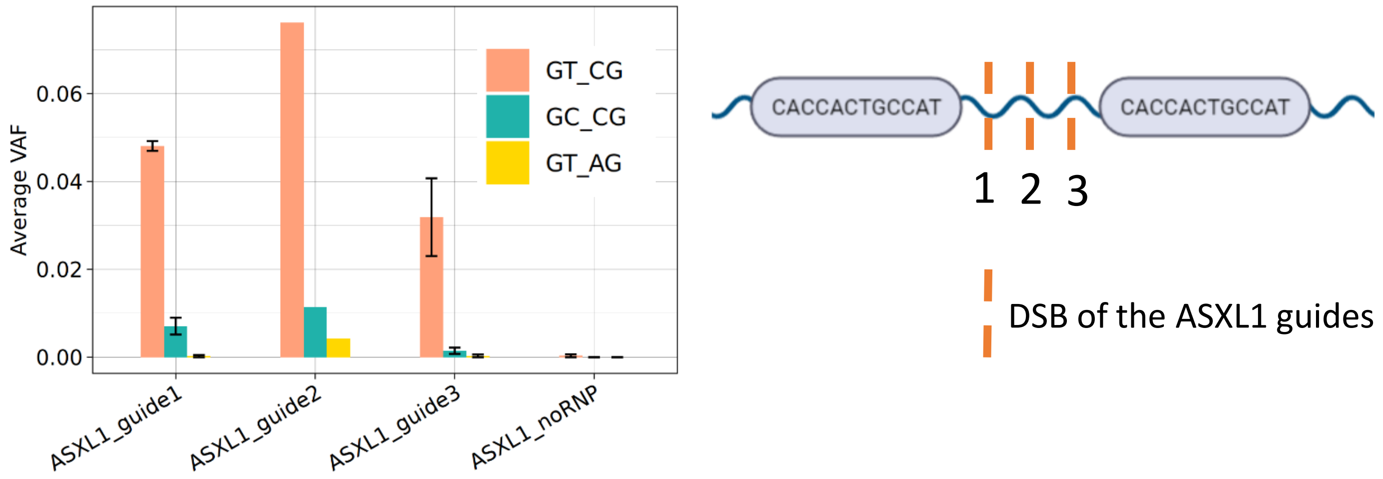

Supplementary Figure S5(A) Plausible Mechanism for Diverse Imperfect Match Deletions. Left panel illustrates the scenario where the homolog is extended towards the right due to mispairing leading to GT_AG flank. Similarly, the right panel represents the simultaneous extension of both flanks of the homolog leading to GC_AG flank. **(B)** Analysis of CRISPR/Cas9 experiments inducing DSBs at various positions between the homologs of *ASXL1*, utilized for the identification of imperfect match deletions

Supplementary Table 1: List of sgRNA guides for inducing DSB to introduce mismatches in the homology arm (Guide 1) and to target DSB formation between the canonical MMEJ homologies (Guide 2).

Supplementary Table 1: CRISPR guides		
Gene	Guide	Sequence
ASXL1	Guide1	CGGCCACCACTGCCATCGGA
ASXL1	Guide2	TCACCACTGCCATAGAGAGG

Supplementary Table 2: Full description of NGS primers to target the *ASXL1* gene

Supplementary Table 2: Targeted sequencing primers for CRISPR experiment				
Gene	Primer orientation	Universal 5' prefix	Target specific sequence	Final primer
ASXL1	Forward	CTACACGACGC TCTTCCGATCT	accctcgcagacattaaagc	CTACACGACGCTCTTCCGATCT accctcgcagacattaaagc
ASXL1	Reverse	CAGACGTGTGC TCTTCCGATCT	gtagatctgacgtacactttc ca	CAGACGTGTGCTCTTCCGATCT gtagatctgacgtacactttcca

Supplementary Table 3: List of guides described in Supplementary Figure S5B

Supplementary Table 3: CRISPR guides		
Gene	Guide	Sequence
ASXL1	Guide1	AGGTCACCACTGCCATAGAG
ASXL1	Guide2	TGGCCGCCTCTCTATGGCAG
ASXL1	Guide3	TCACCACTGCCATAGAGAGG
